# Cost-Effectiveness of “Tele-Square Step Exercise” for Falls Prevention in Fibromyalgia Patients: A Study Protocol

**DOI:** 10.3390/ijerph17030695

**Published:** 2020-01-21

**Authors:** Jorge Carlos-Vivas, Jorge Pérez-Gómez, Serafín Delgado-Gil, José Carlos Campos-López, Manuel Granado-Sánchez, Jorge Rojo-Ramos, Laura Muñoz-Bermejo, Sabina Barrios-Fernandez, María Mendoza-Muñoz, Angelina Prado-Solano, Miguel Ángel Garcia-Gordillo, José Carmelo Adsuar

**Affiliations:** 1 Health, Economy, Motricity and Education Research Group (HEME), Faculty of Sport Sciences, University of Extremadura, Cáceres 10003, Spain; mamendozam@unex.es (M.M.-M.); aprado@unex.es (A.P.-S.); carmelo.adsuar@gmail.com (J.C.A.); 2 Social Impact and Innovation in Health (InHEALTH), University of Extremadura, Cáceres 10003, Spain; serafdel@unex.es (S.D.-G.); ccampos@unex.es (J.C.C.-L.); m_granado@unex.es (M.G.-S.); jorgerr@unex.es (J.R.-R.); lauramunoz@unex.es (L.M.-B.); sabinabarrios@unex.es (S.B.-F.); 3 Department of Terapéutica Médico-Quirúgica, Faculty of Nursing and Occupational Therapy, University of Extremadura, Cáceres 10003, Spain; 4 Facultad de Administración y Negocios, Universidad Autónoma de Chile, sede Talca 3467987, Chile; miguelgarciagordillo@gmail.com

**Keywords:** balance, cognitive aspects, cost-effectiveness, depression, falls prevention, fibromyalgia, happiness, pain, square stepping exercise, strength

## Abstract

Background: Women with fibromyalgia (FM) have 2.5 falls per year compared to the 0.5 falls in people without FM. This fact poses a significant health expense. Square Stepping Exercise (SSE) is a balance training system that has been shown to be effective in preventing falls in the elderly. However, there are neither studies in people with FM nor studies that apply SSE through video-conferencing (Tele-SSE). The objectives of this project are 1) to investigate the applicability, safety, decrease in the number of falls, and incremental cost-effectiveness ratio of prevention of falls program through Tele-SSE in women with FM, and 2) to study the transfer of obtained results to the public and private socio-health economy of Extremadura. Methods/Design: A randomized controlled trial with experimental (Tele-SSE) and control (usual treatment) groups will be carried out. The application of Tele-SSE will be performed for 12 months (three times per week) and one additional follow-up month after the intervention. A focus group including agents to identify key points to transfer the findings to the public and private sectors in Extremadura. One-hundred and eighteen women with FM will be recruited and randomly distributed into the two groups: Experimental (Tele-SSE; *n* = 59) and control group (Usual care; *n* = 59). Primary outcome measures will be: 1) Applicability; 2) safety; 3) annual number of falls; and 4) incremental cost-effectiveness ratio. Secondary outcomes will be: 1) Balance; 2) fear of falling; 3) socio-demographic and clinical information; 4) body composition; 5) physical fitness; 6) physical activity and sedentary behavior; 7) quality of life-related to health, mental health, and positive health; 8) pain; 9) disability level; 10) cognitive aspects; and 11) depressive symptoms. Regarding the focus group, the acceptability of the Tele-SSE will be evaluated in social-sanitary agents and will include Tele-SSE in their services offer. A statistical analysis will be carried out by treatment intention and protocol. In addition, a cost-effectiveness analysis from the perspective of the health system will be performed. Discussion: This project aims to improve the efficiency and equity of physical therapy services based on tele-exercise in preventing falls in people with FM. Furthermore, orientations will be given in order to transfer the obtained findings into the social-sanitary system and market.

## 1. Background

Falls are a major cause of injury and premature death in older people [1]. These falls can cause fractures that negatively affect the quality of life of this population [2], which can pose a great economic expense for public health. For instance, the medical cost of falls of older people supposes an expense of 35 billion of dollars in the United States [3].

Ageing is associated with several changes that are related to a decrease in physical and mental capacities, which increases fragility and the likelihood of falls in older people [4]. Falls and the resulting disability are particularly common among older people [5]. However, disability severity and fall rates may be reduced through exercise interventions [5]. In this regard, several exercise modalities have shown to be effective improving balance in older people, such as strength training [6,7] and/or resistance training [8]. However, levels of adherence to exercise programs in older people are low [9]. For this reason, there are other physical exercise interventions that could be more effective, cheaper, motivating, and specific for preventing falls, such as the so-called Square Stepping Exercise (SSE) [10].

SSE is a program that consists of performing multiple steps in several directions, on a thin carpet divided into 25 × 25 cm squares. SSE may also be done outdoors or indoors. Thus, this is a great alternative to one-way outdoor walking, which is less beneficial in falls prevention and more unsafe for older people [11]. SSE is an alternative intervention that includes physical exercises that is apparently more effective than walking for improving balance and helping to reduce fall risk factors. Moreover, it is recommended as a health promotion exercise in older people [10].

SSE is designed to improve reaction time during the step as well as to restore balance after a stumble. Furthermore, it involves the activation of agonist and antagonist muscles of the lower limbs. The benefit of SSE also extends beyond fall prevention, improving functional ability, lower limb fitness, and health status of the elderly [10,12]. In fact, a recent systematic review showed that SSE is an effective training system in preventing both fall injuries and fear of falls and improves perceived health status in older adults. Moreover, it indicated that SSE may be applied easily, cost-effectively, and in groups [13].

Fibromyalgia (FM) is a chronic syndrome that affects 3.43% of women worldwide, 3.90% in Europe, and 4.2% in Spain [14]. This syndrome has a considerable functional impact on people who suffer from it, as they present chronic pain problems requiring many social, health, and work resources through comprehensive and continuous care [15]. It is estimated that people with FM represent an annual expense of 11,629.03 euros per patient and year in Spain, costing a total of 4223.02 million euros to Spanish health services, which poses a 279.10-euro cost per inhabitant and year [14]. If we consider that, according to the Ministry of Health of Extremadura, a health expenditure of 1549 euros per inhabitant was made in 2016, the expense on fibromyalgia represents 18% of the total health cost per inhabitant in Extremadura. Therefore, any intervention that may help to reduce these percentages could have a positive impact on reducing the costs of the Extremadura health system.

Falls in people with FM amount to 2.5 per year compared to 0.5 for people without FM [16]. Thus, part of the annual health costs of this syndrome comes from falls. The project that we are presenting attempts to reduce costs associated with falls in this population through balance training with the SSE method, conducted by a professional through video-conferencing (Tele-Square Stepping Exercise; Tele-SSE), improving the balance and preventing falls in women with FM. Reviewing the scientific literature, the SSE program appears to improve balance by preventing falls and decreasing the fear of falling in older adults [13], but no publication had studied the benefits of SSE in people with FM.

Therefore, the main objectives of this project are 1) to investigate the applicability, safety, decrease in the number of falls, and incremental cost-effectiveness ratio of a prevention of falls program through Tele-SSE in women with FM, and 2) to identify key factors for its transfer to the public and private social health system, evaluating the acceptability of Tele-SSE in the agents of the social health system in charge of including Tele-SSE in their services offer.

Additionally, this project has a secondary aim to evaluate the effect of Tele-SSE on balance levels, fear of falling, body composition, physical fitness, quality of life related to health and positive health, pain, disability level, depressive symptoms, and cognitive aspects of participants.

## 2. Materials and Methods 

### 2.1. Study Design

The Consolidated Standards of Reporting Trials Statement (CONSORT) methodology for randomized controlled trials [17], as well as the “Recommendations for conduct, methodological practices, and reporting of cost-effectiveness analyses: second panel on cost-effectiveness in health and medicine” [18], were followed. Due to the nature of the intervention, the double-blind study design is not possible because participants will be aware of their group designation, so a single-blind design will be adopted (all assessments will be conducted by research staff who remain unaware of group assignment). A randomized controlled trial will be conducted. Participants will be randomly 1:1 assigned to the intervention group (Tele-SSE) or the control group (usual care).

### 2.2. Ethics Approval

Ethical approval was provided by the Bioethics and Biosafety Committee at the University of Extremadura (approval number: 79/2018) and this study has been registered in the Clinical Trials Registry provided by the Australian New Zealand Clinical Trial Registry (Request number: 378330; https://www.anzctr.org.au/).

### 2.3. Sample Size

The number of participants was calculated based on the change in quality of life assessed using the 5-level Euroqol-5D questionnaire (EQ-5D-5L). As far as we know, there are no available data on the minimum real change in the EQ-5D-5L in fibromyalgia patients, so we have used a reference of pathology that deals with pain, being this real minimum difference of 0.15 [19]. Therefore, a total of 118 participants (Experimental group; *n* = 59 and Control group; *n* = 59) are needed; accepting alpha and beta risks of 0.05 and 0.2, respectively, in a bilateral contrast, to detect a difference equal to or greater than 0.15 units. It is assumed that the common standard deviation of EQ-5D-5L is 0.26, taking as a reference one of the articles by the principal investigator of this project, which evaluated the health-related quality of life through this questionnaire [20]. A follow-up loss rate of 20% has been estimated. 

### 2.4. Randomization and Blinding

After baseline assessments, all participants will be randomly assigned to two groups: Experimental (Tele-SSE) or control (usual care). A simple computer-generated randomization sequence will be created before participants are enrolled (1:1) using the computer software Research Randomizer (Version 4.0, Geoffrey C. Urbaniak and Scott Plous, Middletown, CT, USA) (http://www.randomizer.org). The randomization sequence will be made by a member of the research team without clinical participation in the trial. The assignment will be hidden in a password-protected computer file. Although participants will know their group assignment, outcome evaluators and data analysts will be blinded to the assignment.

### 2.5. Participants

To be included in the project, participants will need to meet the following inclusion criteria:

- Aged between 18 and 66 years old.
- Women diagnosed with FM by a rheumatologist and meeting the more recent diagnostic criteria of the American College of Rheumatology [21]. Thus, (1) pain in at least 4 of 5 regions, (2) symptoms at a similar level for at least 3 months, and (3) widespread pain index (WPI) ≥ 7 and symptom severity scale (SSS) scores ≥ 5 or WPI of 4–6 and SSS score ≥ 9.
- Not suffering pathologies that contraindicate the exercise program or require special attention (coronary, thrombosis, bone, renal, moderate or severe pulmonary pathologies, etc.). The Physical Activity Readiness questionnaire (PAR-Q) for the practice of physical activity and sport [22] will be administered to control if anyone suffers any disease that impedes the physical load.
- Not suffering any pathology that requires the use of psychotropics or affects the vestibular system to avoid possible influences on balance measures.
- Not suffering any neurodegenerative pathology such as: Parkinson’s disease or multiple sclerosis.
- Returning the signed informed consent form of the study.
- Not being physically active as defined by the World Health Organization (WHO) during the previous 3 months.
- Trying to keep in the same FM association.
- Having the ability to walk and communicate on her own.
- Accessing to a device where video-conferences can be held.

### 2.6. Interventions

**Experimental group (Tele-SSE):** The experimental group will receive Tele-SSE training, 3 times a week, for 12 months. They will be given training guidelines through a brochure and all sessions will be tracked through video-conferencing on a mobile phone. Two to six patients will simultaneously perform the exercise in a common place, which will be a room or dependency yielded by each participating association with Internet connection via WIFI. SSE will be carried out on a 250 × 100 cm dimensioned thin carpet, divided into 40 squares of 25 × 25 cm. SSE includes a total of 200 different movement patterns that are classified, based on its difficulty, into three general levels: Beginner, intermediate, and advanced. The beginner level has two sublevels, while the intermediate and advanced levels have 3 sublevels. The proposed intervention will include a progression along with the different levels, which is shown in Table 1. Participants will start by movement patterns like walking and little by little they will make more complex patterns that will require multidirectional movements. Participants should not step on the squares dividing lines. The number of steps patterns and difficulty will monthly increase until the maximum level, where they will be maintained until the end of the intervention.

In all sessions, the SSE expert will phone the group of participants and will indicate the training to be done. Participants will have a brochure detailing the 200 movement patterns, which they can consult while the SSE expert indicates the sequence to follow during the class that day. Before starting the session, the first activity to be carried out will be to remember the last movement pattern executed in the previous session. Once the patients have memorized the pattern, which is expected to be achieved after 4–5 repetitions, based on a previous study [23], they will continue with the session autonomously. At the end of each session, they will contact the SSE expert who will ask them about the session development and will question them about two scales: The rating will be of perceived exertion [24] and level of pain, also recording any incident or problem during the training session.

**Control group:** The control group will continue with the treatment carried out within the public health system (usual care).

### 2.7. Measures and Procedures

A variety of tools will be used to assess the utility and effectiveness of the exercise program (Table 2).

#### 2.7.1. Randomized Trial Main Measures

**Applicability.** The percentage of participants with FM who can perform the proposed training. If any participant is unable to perform the intervention, the cause will be noted.

**Safety.** A record shall be kept about any incidence, injury or problem during the sessions, as well as if possible, the origin of the problem.

**Number of falls in the last year and six months.** Participants will be asked about the number of falls that they have suffered in the past year and six months. The World Health Organization (WHO) definition of falls will be adopted, namely: “Involuntary events that cause the body to lose its balance and find itself on land or on another firm surface that stops it”.

**Incremental cost-effectiveness ratio.** The average cost of the intervention and the effects on health will be calculated for both intervention and control groups. The measurement of health effects is defined as Quality Adjusted Life Years (QALYs), which is calculated by multiplying the life years (life expectancy) with the quality of life of the participants [25]. The health-related quality of life will be evaluated using the EQ-5D-5L questionnaire [26] and this measure will be used for sample calculation. Incremental cost-effectiveness ratio will be calculated by dividing the difference between the average costs of both groups by the difference in mean QALYs gained in both groups. The utilities needed to calculate the QALYs will be computed for each set of health status using the algorithm available on the official website of the EuroQol group (http://www.euroqol.org/). The algorithm used to calculate the EQ-5D-5L utility index will be the “crosswalking” of the EuroQol levels Spanish version. This utility index is comprised between −0.654 (corresponding to the worst state of health and equivalent to 55555) and 1 (perfect state of health and equivalent to 11111).

#### 2.7.2. Focus Group Main Measure

Once the randomized controlled trial has been carried out, and considering its preliminary results, a focus group will be set up around questions previously drawn up by the research group, in order to determine the acceptability of Tele-SSE in the social-sanitary system agents. Key factors will also be identified for its transfer to the public and private social-sanitary system. The transcripts will be analyzed with the support of specific software for discourse analysis, which will be used to organize and classify the data into categories and then proceed to order and analyze them [27].

#### 2.7.3. Secondary Measures

##### Sociodemographic data

Participants will be asked about her age, income, disease time of diagnosis, educational level, marital status...

##### Balance

*Time Up and Go Test (TUG).* Participants will carry out three variants of this test based on the covered distances: 2.40 m [28], 3 m [29], and 10 m [30]. This test has been shown to be valid and reliable (ICC = 0.93, SEM = 0.58, MDC = 1.60) [31]. Each participant will start seated on a chair with her arms and trunk supported. The participant will be instructed to stand with the word “go” and walk in a straight line for 2.40 m, 3 m, and 10 m (depending on the test variant), turn 180°, and walk back to the chair to sit on it again. Prior to recording the measurements, a familiarization test will be conducted. After 1-minute rest, the test will be performed twice, separated by the 1-minute rest. Time will be measured from the standing start movement until the participant sits on the chair again, leaning her back on the support of the chair. The best attempts of each variant will be used for analysis. 

*One-leg stance.* Two variants of this test will be performed: Eyes open (ICC = 0.99) and eyes closed (ICC = 0.95). The test consists of timing the time that the person can be in balance on one leg with arms crossed on the upper train. The test begins when the foot is raised from the ground and ends in the following assumptions: 1) When the arms are uncrossed, 2) when the raised foot touches the ground, 3) when the supported foot moves, 4) when more than 45 seconds have elapsed, and 5) when the eyes are opened in the test with closed eyes. The procedure will be repeated 3 times and each time recorded on the data collection sheet [32,33].

*Four square steps test (FSST).* Four squares will be formed by placing adhesive tape on a flat floor. Each square will be numbered. At the beginning of the test, patients who will be standing on square number 1 opposite square number 2 will be asked to walk as fast as possible on each square consecutively (1-2-3-4-4-3-2-1) without touching the gaps and with both feet contacting the floor. The time needed to complete the complete cycle will be taken for analysis. This test has been shown valid and reliable (ICC = 0.98) [34]. 

*Berg Balance Scale (BBS).* The BBS will be used to evaluate the dynamic balance. The BBS consists of 14 elements that evaluate daily performance (ICC = 0.98). Each activity level of competence is graded on a scale of 5 points ranging from 0 (unable to do) to 4 (able to do independently and safely). Each functional parameter will be individually explained and demonstrated to the patient. Participants will be asked to perform all parameters; the total score will be calculated as the sum of the scores obtained from each parameter. The maximum score that can be obtained in the test is 56. The different threshold for this test is classified as: A high risk of falling (0–20 points), a moderate risk of falling (21–40 points), and a low risk of falling (41–56 points) [35].

*Activity-Specific Balance Confidence Scale (ABC).* This scale consists of a rating of 16 questions for evaluating the participants’ balance confidence in daily activities between 0 and 100. Patients scoring above 67% will be considered at high risk for falls. The Spanish version of the ABC scale will be used [35].

##### Fall Risk

*The 21-item Fall Risk Index (FRI-21)*. This questionnaire consists of 21 items: Presence of stumbling, ability to climb stairs, decreased walking speed, ability to cross the road within the green signal interval, ability to walk 1 km continuously, ability to stand on one leg for 5 s, use of a cane, ability to squeeze a towel, dizziness, back bent, knee pain, vision problem, hearing problem, forgetfulness, anxiety about falls, use of more than five prescription medications, sensation of darkness at home, presence of obstacles inside the house, presence of barriers on the floor, daily stair use at home, and steep slopes near the house. Each item receives a score of 1 (risk) or 0 (no risk), and the sum of all items ranged from 0 (low fall risk) to 21 (high fall risk), with higher scores indicating higher risk of falls. A cut-off point of 9–10 on the 21-item FRI-21 is useful for early detection of fall risk [36].

##### Fear of Falling

The fear of falling will be measured using the Falls Efficacy Scale-International (FES-I) questionnaire, developed and validated by the Prevention of Falls Network Europe (ProFaNE). This questionnaire has become a widely accepted tool for assessing fear of falling [37] and has excellent reliability and validity (ICC = 0.96) [37] in different cultures and languages [38], including in the Spanish language [39]. The FES-I is a self-reported questionnaire that provides information about the level of concern of falls in a range of activities of daily living. The original questionnaire contains 16 items and is graded on a four-point scale (1 = not very concerned to 4 = very concerned). Therefore, the best possible value is 16 and the worst is 64. It will also be evaluated through a Visual Analogue Scale for Fear of Falling (VAS-FOF) (ICC = 0.57) [40].

##### Body Composition

Height and weight will be measured using a stadiometer (Seca 22, Hamburg, Germany). Also, the waist and hip circumferences will be evaluated, with participants in a standing position (Harpenden Anthropometric Tape, Holtain Ltd). Moreover, a bioimpedanciometer (TANITA MC 780MA) will be used to evaluate fat mass, free-fat mass, lean mass, bone mass, and muscle mass index [41].

##### Physical Condition

*The 6-minute Walking Test*. This test measures the maximum distance that each participant can walk in 6 min along a 45.7-meter rectangular course [29]. This test has been shown valid and reliable (ICC = 0.92, SEM = 23.52, MDC = 65.20) [31].

*Lower-Body Strength*. The 30-second Chair Stand Test (ICC = 0.91, SEM = 0.91, MDC = 2.52) [31] consists of counting the number of times that the participant can get up completely from a sitting position with a straight back and flat feet on the floor, without pushing with the arms within the 30 s [42].

*Upper-Body Strength.* This test consists of determining the number of times you can lift a weight by performing an arm flexo-extension with a hand weight of 2.3 kg for women for 30 seconds [42]. This test has been shown to be valid and reliable (ICC = 0.92, SEM = 1.14, MDC = 3.16) [31]. Also, a handgrip test (ICC = 0.95, SEM = 1.46, MDC = 4.04) [31] will be performed using a digital dynamometer (TKK 5101 Grip-D; Takey, Tokyo, Japan) [43]. Participants will perform (alternately with both hands) both tests twice. The best value of 2 tests will be chosen for each hand and the average of both hands will be used for analysis.

*Muscle Trunk Strength-Endurance*. This measure will be assessed using a test of abdominal and trunk muscle resistance on a mat (r > 0.90) [44]. To evaluate the strength of the flexor muscle of the trunk, participants are asked to lie supine and raise their lower limbs with 90 flexion degrees on hip and knee and; in order to evaluate the resistance of the extensor muscles, patients lie down in pronation. In both cases, participants are asked to move their shoulders away from the ground by bending or extending the trunk. They should hold this position as long as possible, without exceeding 5 min [43].

*Upper-Limb Flexibility*. The Back-Scratch Test will be carried out (ICC = 0.96, SEM = 2.77, MDC = 7.68) [31], which is a measure of the total range of movement of the shoulder and involves measuring the distance between (or superposition of) the middle fingers behind the back with a ruler. The best score of two attempts will be recorded for each arm and the average of both arms will be used for analyses [42].

*Lower-Limb Flexibility.* The Chair Sit and Reach Test will be used (ICC = 0.94, SEM = 3.13, MDC = 8.66) [31]. Patients start from a sitting position with one leg extended. Then, they lean slowly by sliding their hands across the extended leg to touch (or pass) their toes. The number of centimeters will be recorded [45]. Two trials shall be measured for each leg and the best value achieved each leg shall be recorded. The average of both legs shall be used for analyses.

*Velocity.* The Brisk Walking Test shall be used (ICC = 0.93). This test consists of measuring the time that each participant spends walking 30 m. There will be 2 repetitions with 1-minute rest between them. The best result will be used for analysis [46].

*Functional scope.* The Functional Reach Test will be used (ICC = 0.92) [47]. The participant will be placed next to a wall with arms at 90 degrees from the trunk that will have to reach the maximum frontal distance and remaining in that position during a few seconds, without altering its support base. The maximum distance achieved perpendicularly to the wall will be recorded.

*Short Physical Performance Battery (SPPB).* The battery is composed of 3 direct observation tests: Walking velocity, balance, and time to get up 5 times from a chair [48].

*Self-perceived physical fitness.* The International Fitness Scale (IFIS) will be applied (Kappa = 0.45) [49]. This instrument consists of 5 Likert scale questions about how participants perceive their overall physical fitness, cardio-respiratory fitness, muscle strength, speed-agility, and flexibility. Response options are: “Very poor”, “poor”, “average”, “good”, and “very good” compared to their friends.

##### Pain

Pain will be evaluated by pressing on the different trigger points. This will require an algometer that can evaluate the pressure exerted on these points, such as the Force Ten FDX (Wagner Instrument). The evaluator will have undergone measurement training prior to the initial measurements [50]. In addition, each participant will be asked about their pain using an Analog Visual Pain Scale [51,52] at baseline, at the end of the treatment and follow up; as well as before and after each training.

##### Health-Related Quality of Life (HRQoL)

Health-related Quality of Life will be evaluated using the following questionnaires:

*15-D.* A 15-dimensional questionnaire (Cronbach’s alpha = 0.79) with 5 degrees of response in each dimension. For its result, it contains a final measure from 0 to 1, where 0 is the worst possible quality of life and 1 is the best. This questionnaire is based on preferences for what allows cost-effectiveness analysis [53,54].

*SF-12.* A 12-question tool (Cronbach’s alpha > 0.70), an abbreviated version of the SF-36 questionnaire, results in 8 dimensions (physical function, physical role, body pain, general health, vitality, social function, emotional role, mental health) and 2 summary components (physical and mental). These dimensions, as well as the components, score from 0 to 100, where 0 is the worst state of health and 100 the best. This questionnaire allows the obtainment of a utility index, including the SF-6D, which is contained within it. The index from SF-6D is comprised between 0 and 1, where 0 is the worst possible state of health and 1 the best [55].

*HUIv3.* This questionnaire consists of 8 dimensions with 5 or 6 possible response levels depending on the dimension (Cronbach’s alpha = 0.79). The dimensions are: Vision, hearing, speech, wandering, dexterity, emotion, cognition, and pain. The score ranges from 0 to 1, with 0 being the worst possible state of health and 1 being the best [56]. The Verran Snyder–Halpern sleep scale [57] will also be used, as well as a visual analogue sleep scale [58].

##### Incapacity in Fibromyalgia

Two different questionnaires will be applied: The Fibromyalgia Impact Questionnaire (FIQ) and the Revised Fibromyalgia Impact Questionnaire (FIQ-R).

*Fibromyalgia Impact Questionnaire (FIQ).* This questionnaire consists of 20 questions, the first 10 belonging to a single dimension. The total score of the questionnaire is between 0 and 100, with 0 being no disability due to FM and 100 being the maximum disability due to FM [59].

*Revised Fibromyalgia Impact Questionnaire (FIQ-R)* [60,61]. This questionnaire contains 21 items that mention symptoms or problems you have had in the last 7 days with a response scale ranging from 0 to 10, where 0 is the absence of the problem and 10 is the greatest intensity of the problem. The FIQ-R consists of 3 domains: “Function” (first 9 items), “Global Impact” (two items), and “Severity of symptoms” (10 items). The main difference with the FIQ is that the third domain includes 4 new aspects related to memory, sensitivity, balance, and sensitivity to loud noises, bright lights, smells, and cold temperatures. In addition, the scoring system also has several advantages. This questionnaire has been shown valid and reliable (Cronbach’s alpha = 0.93) [60].

##### Depression

The depressive symptoms of FM patients will be evaluated as one of the commonly associated symptoms. The Beck Depression Inventory second edition (BDI-II) is a 21-item self-report instrument designed to assess the severity of depressive symptomatology in adults and adolescents, with a minimum age of 13 years (Cronbach’s alpha = 0.94) [62]. In each of the items, the person must choose, from a set of four alternatives ordered from least to most severe, the sentence that best describes his/her state during the last two weeks, including the day when he/she completes the instrument. In terms of correction, each item is scored from 0 to 3 points depending on the alternative chosen and, after directly adding up the score of each item, a total score ranging from 0 to 63 can be obtained. Sometimes it is the case that the person chooses more than one alternative in an item given. In this case, the punctuation of the chosen phrase is taken more seriously. Several studies on psychometrics guarantee the reliability and validity of the BDI-II in very diverse samples [62,63].

##### Happiness

Happiness will be assessed using the General Happiness Scale (Cronbach’s alpha = 0.86) [64] and the Satisfaction with Life Scale (SWLS) (Cronbach’s alpha = 0.78) [65], which will be applied before, during, and after treatment.

##### Physical Activity and Sedentary Behaviors

*Physical activity in leisure time.* The Leisure Time Physical Activity Instrument (LTPAI) will be used to assess the physical activity performed by participants in their leisure time (ICC = 0.84) [66]. This questionnaire consists of 4 items with 3 activity levels: Light, moderate, and vigorous (a short description of each category will be presented). Subjects will be asked to recall the average number of hours a week during the previous 4 weeks that they had spent engaged in physical activity and at what activity level. The scale will be simplified into the following 3 levels: (1) 0.5 to 1.5 h a week, (2) 2 to 4 h a week, and (3) more than 4 h a week, and the respondent will be asked to provide answers in hours. For the first 2 activity levels, the mean number of hours (1 and 3 h, respectively) will be used to calculate the total score. If no level will be selected for a category, the number of hours will be assigned a value of 0 for that category. The number of hours will indicate by the subjects for each intensity category will be summed to obtain the leisure-time physical activity level for 1 week [66].

*Physical Activity at Home and Work.* The Physical Activity at Home and Work Instrument (PAHWI) will be used (ICC = 0.87) [66]. This questionnaire consists of 7 items with 3 categories for work performed at home (light, moderate, heavy activity) and 4 categories for employment (sedentary, light, moderate, heavy activity). A short description of each category will be presented, and the respondents will be asked to report the amount of time spent performing each of the activity categories. The hours for each category will be summed to obtain the total score for the PAHWI (activity at housework and activity at the workplace) [66].

*Sedentary Behavior Questionnaire (SBQ).* This test assesses the amount of time spent on 11 behaviors (ICC = 0.85) [67]. The 11 items will be completed separately for weekdays and weekend days. Response options are: “None”, “15 min or less”, “30 min”, “1 hour”, “2 h”, “3 h”, “4 h”, “5 h”, or “6 h or more”. The time spent on each behavior will be converted into h (e.g., a response of 15 min will be recorded as 0.25 h) [67,68]. For the total scores of sedentary behaviors, hours per day for each item will be summed separately for weekday and weekend days. To obtain weekly estimates, weekday hours will be multiplied by 5 and weekend hours will be multiplied by 2, and these will be summed for total hours/week. For the summary variables of total hours/day spent in sedentary behaviors (weekday and weekend) and total sedentary hours/week, responses higher than 24 h/day will be truncated to 24 h/day.

##### Clinical Global Impression

Improvement due to interventions will be measured using the Patient Global Impression of Improvement Scale [69], which uses the Likert scale.

##### Tests Familiarization and Reliability

Before each session, there will be a warm-up phase where the participant will have the test procedure explained, including the specific test trial, before it will be carried out.

The different tests inter-session reliability will be evaluated by repeating the tests two weeks after with part of the sample participants. All possible tests will be carried out under single and dual-tasks conditions.

### 2.8. Statistical Analysis

Baseline characteristics of participants will be presented as the mean (standard deviation) for continuous variables and proportions for categorical variables. The normality will be checked using the Kolmogorov-Smirnov test. Then, two types of analyses will be performed: 1) An intention-to-treat analysis including all participants and 2) a per-protocol analysis using only those participants who complete the study.

**Intention-to-treat analysis.** This analysis will include all randomly assigned participants in their corresponding groups. Multiple imputations will be used to impute lost data. The effects of the intervention on the main and secondary variables will be computed using a repeated measures ANCOVA test, adjusted by age and baseline values. Results shall include the Cohen’s *d* effect size (95% confidence interval) and statistical significance for each dependent variable respect to time and its interaction effects (group × time). Statistical significance shall be established at the conventional level of *p* < 0.05. In sensitivity analyses, the data imputation will be made from the baseline patient data and from those participants who completed the study, in order to avoid estimation biases. 

**Analysis by protocol.** Similar analyses than those described above will be carried out, but only using participants who have attended at least 75% of the sessions.

### 2.9. Cost-Effectiveness Analysis

A cost-utility analysis will be conducted with a health system perspective in line with the methodological recommendations of health economists [70], and the guidelines defined in the scientific literature [18].

The study will be carried out considering the costs and health effects of the intervention, including the effect of Tele-SSE on the number of falls. Only the direct costs of the program (i.e. medication, primary care visits, and hospital admissions) will be considered. Likewise, the salaries of the person hired to carry out the video-conferences will be considered in agreement with the set-out guidelines, based on the corresponding collective agreement. It will also include the costs of the essential material for the Tele-SSE implementation, such as training mats, mobile phones, and Internet connection.

First, the average costs and effectiveness will be calculated for each group, i.e., the Quality Adjusted Life Years (QALYs) earned. Subsequently, the incremental cost-effectiveness ratio will be calculated [71] and various sensitivity analyses will be conducted, including probabilistic, which will be carried out with 1000 repetitions [72]. These replicas will be included in the cost-effectiveness plan and will be used to make the acceptability curve. Then, we will be able to see in which quadrant of the cost-effectiveness plan the Tele-SSE intervention is located, which will indicate whether the Tele-SSE has been costly and effective than the intervention made by the control group (usual care).

## 3. Discussion

This project would be the first that uses the Tele-SSE training system for falls prevention in fibromyalgia people and, to our knowledge, the first study carried out in Spain using the SSE training system. If the effectiveness of this treatment for falls prevention and its cost-effectiveness compared to conventional treatment (usual care) is demonstrated, this would pose an economic saving opportunity to the health system. It would also help to implement this type of methodology in the health system, not only for fibromyalgia patients, but also for people with an increased risk of falls, such as frail elderly; a population where the efficacy of SSE training has recently been shown [13]. It would also have great repercussions, since ageing of the population, according to all statistical projections, will increase in the next years [73,74].

In addition, to our knowledge, this would be the first study that would show the cost-effectiveness of Tele-SSE. Thus, if the effectiveness and cost-effectiveness of this training system are proven, it could be applied by several agents, which could be interested in taking advantage of the benefits of these types of activities. It should be assumed that this training method does not require any specific installation and may be carried out indoors or outdoors. Therefore, due to this being a low-cost healthcare technology that can be easily standardized by levels of difficulty (see methodology), its implementation in both public and private sectors would not pose any problems.

Moreover, the main measure of the additional focus group study proposed will revolve around investigating the acceptability of Tele-SSE in the agents of the social-sanitary system. Thus, key factors will also be identified for its transfer to the public and private social-sanitary system. Regarding the public sector, after a focus group with the main health policy decision-makers, where the applicability and acceptability of this health technology would be analyzed, these decision-makers would be urged to include the Tele-SSE within the recommendations offered by health professionals to fibromyalgia people for better management of the disease. Furthermore, we would encourage the application of Tele-SSE or SSE within the services offered by public health programs, such as the so-called “Exercise Looks After you” [75], increasing its cost-effectiveness in everything related to falls prevention in fibromyalgia patients, and even being extrapolated to other populations with increased risk of falls, such as frail elderly. Likewise, the possibility of implementing this training system in different associations of diseases associated with high risk of falls (e.g., fibromyalgia, multiple sclerosis, Parkinson’s disease) and at home (e.g., for elderly) would be studied. In the private sector, the focus group study would include the heads of health and sports centers, focusing on the applicability of Tele-SSE in their centers and showing the results of the study in order to highlight the potential advantages of applying Tele-SSE or SSE in the private sector. Also, meetings would also be held with heads of insurance companies to see the possibility of including discounts or life insurance with special conditions for those people who regularly carry out Tele-SSE training.

## 4. Conclusions

This project will investigate the applicability, safety, decrease in the number of falls, and incremental cost-effectiveness ratio of a 12-month Tele-SSE prevention of falls program in women with fibromyalgia, as well as, the transfer of obtained results to the public and private socio-health economy of Extremadura will be studied. Results of this investigation will help to improve the efficiency and equity of physical therapy services based on tele-exercise in preventing falls in people with fibromyalgia. Furthermore, orientations will be proposed in order to transfer the obtained findings into the social-sanitary system and market. If the interventions proved to be effective and safe, this study would provide feasible and low-cost alternatives for health professionals in the management and treatment of fibromyalgia.

## Figures and Tables

**Table 1:** Proposal progression of Tele-SSE training.

Month	Frequency (Days a Week)	Session Duration	Number of Steps per Sequence	Level of Difficulty
1	3	50 min	4	Beginner 1 and 2
2	3	50 min	6	Intermediate 1
3	3	50 min	6	Intermediate 2
4	3	50 min	8	Intermediate 3
5	3	50 min	8	Advanced 1
6	3	50 min	8	Advanced 2
7	3	50 min	8	Advanced 3
8	3	50 min	8	Advanced 3
9	3	50 min	8	Advanced 3
10	3	50 min	8	Advanced 3
11	3	50 min	8	Advanced 3
12	3	50 min	8	Advanced 3

**Table 2:** Assessments schedule for both experimental and control group.

Assessment	Baseline	Month 3	Month 6	Month 9	Month 12	Month 13
Applicability	x				x	x
Safety	x				x	x
Number of falls in the last year and six months	x		x		x	x
Incremental cost-effectiveness ratio	x				x	x
Sociodemographic data	x					
Balance	x	x	x	x	x	x
Fall Risk	x	x	x	x	x	x
Fear of falling	x	x	x	x	x	x
Body composition	x	x	x	x	x	x
Physical condition	x	x	x	x	x	x
Pain	x	x	x	x	x	x
Health-related quality of life	x	x	x	x	x	x
Incapacity of fibromyalgia	x	x	x	x	x	x
Depression	x	x	x	x	x	x
Happiness	x	x	x	x	x	x
Physical activity and sedentary behaviors	x	x	x	x	x	x
Clinical global impression	x	x	x	x	x	x

## References

[1] Burns E., Kakara R. (2018). Deaths from falls among persons aged ≥ 65 years—United States, 2007–2016. MMWR Morb. Mortal. Wkly..

[2] Román E., Córdoba J., Torrens M., Guarner C., Soriano G. (2013). Falls and cognitive dysfunction impair health-related quality of life in patients with cirrhosis. Eur. J. Gastroenterol. Hepatol..

[3] Houry D., Florence C., Baldwin G., Stevens J., McClure R. (2016). The CDC Injury Center’s response to the growing public health problem of falls among older adults. Am. J. Lifestyle Med..

[4] Rubenstein L.Z. (2006). Falls in older people: Epidemiology, risk factors and strategies for prevention. Age Ageing.

[5] Sherrington C., Lord S.R., Vogler C.M., Close J.C., Howard K., Dean C.M., Clemson L., Barraclough E., Ramsay E., D O’Rourke S. (2009). Minimising disability and falls in older people through a post-hospital exercise program: A protocol for a randomised controlled trial and economic evaluation. BMC Geriatr..

[6] Silveira P., Van De Langenberg R., Van Het Reve E., Daniel F., Casati F., De Bruin E.D. (2013). Tablet-based strength-balance training to motivate and improve adherence to exercise in independently living older people: A phase II preclinical exploratory trial. J. Med. Internet Res..

[7] van Het Reve E., Silveira P., Daniel F., Casati F., De Bruin E.D. (2014). Tablet-based strength-balance training to motivate and improve adherence to exercise in independently living older people: Part 2 of a phase II preclinical exploratory trial. J. Med. Internet Res..

[8] Virág A., Karóczi C., Jakab A., Vass Z., Kovacs E., Gondos T. (2015). Short-term and long-term effects of nordic walking training on balance, functional mobility, muscle strength and aerobic endurance among Hungarian community-living older people: A feasibility study. J. Sport Med. Phys. Fit..

[9] Nelson M.E., Rejeski W.J., Blair S.N., Duncan P.W., Judge J.O., King A.C., Macera C.A., Castaneda-Sceppa C. (2007). Physical activity and public health in older adults: Recommendation from the American College of Sports Medicine and the American Heart Association. Circulation.

[10] Shigematsu R., Okura T., Nakagaichi M., Tanaka K., Sakai T., Kitazumi S., Rantanen T. (2008). Square-stepping exercise and fall risk factors in older adults: A single-blind, randomized controlled trial. J. Gerontol. A Biol. Sci. Med. Sci..

[11] McClure R.J., Turner C., Peel N., Spinks A., Eakin E., Hughes K. (2005). Population-based interventions for the prevention of fall-related injuries in older people. Cochrane Database Syst. Rev..

[12] Shigematsu R., Okura T., Sakai T., Rantanen T. (2008). Square-stepping exercise versus strength and balance training for fall risk factors. Aging Clin. Exp. Res..

[13] Fisseha B., Janakiraman B., Yitayeh A., Ravichandran H. (2017). Effect of square stepping exercise for older adults to prevent fall and injury related to fall: Systematic review and meta-analysis of current evidences. J. Exerc. Rehabil..

[14] Cabo A., Cerdá G., Trillo J. (2017). Fibromyalgia: Prevalence, Epidemiologic Profiles and Economic Costs. Med. Clin..

[15] Carville S.F., Arendt-Nielsen S., Bliddal H., Blotman F., Branco J., Buskila D., Da Silva J., Danneskiold-Samsøe B., Dincer F., Henriksson C. (2008). EULAR evidence-based recommendations for the management of fibromyalgia syndrome. Ann. Rheum. Dis..

[16] Meireles S.A., Antero D.C., Kulczycki M.M., Skare T.L. (2014). Prevalence of falls in fibromyalgia patients. Acta Ortop. Bras..

[17] Schulz K.F., Altman D.G., Moher D. (2010). CONSORT 2010 statement: Updated guidelines for reporting parallel group randomised trials. BMC Med..

[18] Sanders G.D., Neumann P.J., Basu A., Brock D.W., Feeny D., Krahn M., Kuntz K.M., Meltzer D.O., Owens D.K., Prosser L.A. (2016). Recommendations for conduct, methodological practices, and reporting of cost-effectiveness analyses: Second panel on cost-effectiveness in health and medicine. JAMA.

[19] Parker S.L., Adogwa O., Paul A.R., Anderson W.N., Aaronson O., Cheng J.S., McGirt M.J. (2011). Utility of minimum clinically important difference in assessing pain, disability, and health state after transforaminal lumbar interbody fusion for degenerative lumbar spondylolisthesis. J. Neurosurg..

[20] Collado-Mateo D., Chen G., Garcia-Gordillo M.A., Iezzi A., Adsuar J.C., Olivares P.R., Gusi N. (2017). Fibromyalgia and quality of life: Mapping the revised fibromyalgia impact questionnaire to the preference-based instruments. Health Qual. Life Outcomes.

[21] Wolfe F., Clauw D.J., Fitzcharles M.-A., Goldenberg D.L., Häuser W., Katz R.L., Mease P.J., Russell A.S., Russell I.J., Walitt B. (2016). 2016 Revisions to the 2010/2011 fibromyalgia diagnostic criteria. Semin. Arthritis Rheum..

[22] Cardinal B.J., Esters J., Cardinal M.K. (1996). Evaluation of the revised physical activity readiness questionnaire in older adults. Med. Sci. Sports Exerc..

[23] Shigematsu R., Okura T., Nakagaichi M., Nakata Y. (2014). Effects of exercise program requiring attention, memory and imitation on cognitive function in elderly persons: A non-randomized pilot study. J. Gerontol. Geriatr. Res..

[24] Andrade C.P., Zamunér A.R., Forti M., de França T.F., da Silva E. (2017). A escala CR-10 de Borg é viável para quantificar a intensidade do exercício aeróbio em mulheres com síndrome fibromiálgica. Fisioterapia e Pesquisa.

[25] Mehrez A., Gafni A. (1989). Quality-adjusted life years, utility theory, and healthy-years equivalents. Med. Decis. Mak..

[26] Herdman M., Gudex C., Lloyd A., Janssen M., Kind P., Parkin D., Bonsel G., Badia X. (2011). Development and preliminary testing of the new five-level version of EQ-5D (EQ-5D-5L). Qual. Life Res..

[27] Flick U. (2018). An Introduction to Qualitative Research.

[28] Rose D.J., Jones C.J., Lucchese N. (2002). Predicting the probability of falls in community-residing older adults using the 8-foot up-and-go: A new measure of functional mobility. J. Aging Phys. Act..

[29] Spagnuolo D.L., Juergensen S.P., Iwama A.M., Dourado V.Z. (2010). Walking for the assessment of balance in healthy subjects older than 40 years. Gerontology.

[30] Evans T., Jefferson A., Byrnes M., Walters S., Ghosh S., Mastaglia F.L., Power B., Anderton R.S. (2017). Extended “Timed Up and Go” assessment as a clinical indicator of cognitive state in Parkinson’s disease. J. Neurol. Sci..

[31] Carbonell-Baeza A., Alvarez-Gallardo I., Segura-Jiménez V., Castro-Pinero J., Ruiz J., Delgado-Fernández M., Aparicio V. (2015). Reliability and feasibility of physical fitness tests in female fibromyalgia patients. Int. J. Sports Med..

[32] Springer B.A., Marin R., Cyhan T., Roberts H., Gill N.W. (2007). Normative values for the unipedal stance test with eyes open and closed. J. Geriatr. Phys. Ther..

[33] Vellas B.J., Wayne S.J., Romero L., Baumgartner R.N., Rubenstein L.Z., Garry P.J. (1997). One-leg balance is an important predictor of injurious falls in older persons. J. Am. Geriatr. Soc..

[34] Dite W., Temple V.A. (2002). A clinical test of stepping and change of direction to identify multiple falling older adults. Arch. Phys. Med. Rehab..

[35] Berg K.O., Wood-Dauphinee S.L., Williams J.I., Maki B. (1992). Measuring balance in the elderly: Validation of an instrument. Can. J. Public Health.

[36] Ishimoto Y., Wada T., Kasahara Y., Kimura Y., Fukutomi E., Chen W., Hirosaki M., Nakatsuka M., Fujisawa M., Sakamoto R. (2012). Fall Risk Index predicts functional decline regardless of fall experiences among community-dwelling elderly. Geriatr. Gerontol. Int..

[37] Yardley L., Beyer N., Hauer K., Kempen G., Piot-Ziegler C., Todd C. (2005). Development and initial validation of the Falls Efficacy Scale-International (FES-I). Age Ageing.

[38] Kempen G.I., Todd C.J., Van Haastregt J.C., Rixt Zijlstra G.A., Beyer N., Freiberger E., Hauer K.A., Piot-Ziegler C., Yardley L. (2007). Cross-cultural validation of the Falls Efficacy Scale International (FES-I) in older people: Results from Germany, the Netherlands and the UK were satisfactory. Disabil. Rehabil..

[39] Lomas-Vega R., Hita-Contreras F., Mendoza N., Martínez-Amat A. (2012). Cross-cultural adaptation and validation of the Falls Efficacy Scale International in Spanish postmenopausal women. Menopause.

[40] Scheffer A.C., Schuurmans M.J., VanDijk N., Van Der Hooft T., De Rooij S.E. (2010). Reliability and validity of the visual analogue scale for fear of falling in older persons. J. Am. Geriatr. Soc..

[41] NIH (1996). Bioelectrical impedance analysis in body composition measurements: National Institutes of Health Technology Assessment Conference Statement. Am. J. Clin. Nutr..

[42] Rikli R.E., Jones C.J. (1999). Development and validation of a functional fitness test for community-residing older adults. J. Aging Phys. Act..

[43] Ruiz-Ruiz J., Mesa J.L., Gutiérrez A., Castillo M.J. (2002). Hand size influences optimal grip span in women but not in men. J. Hand Surg..

[44] Ito T., Shirado O., Suzuki H., Takahashi M., Kaneda K., Strax T.E. (1996). Lumbar trunk muscle endurance testing: An inexpensive alternative to a machine for evaluation. Arch. Phys. Med. Rehab..

[45] Jones C.J., Rikli R.E., Max J., Noffal G. (1998). The reliability and validity of a chair sit-and-reach test as a measure of hamstring flexibility in older adults. Res. Q. Exerc. Sport.

[46] Andersson M., Moberg L., Svantesson U., Sundbom A., Johansson H., Emtner M. (2011). Measuring walking speed in COPD: Test-retest reliability of the 30-metre walk test and comparison with the 6-minute walk test. Prim. Care Respir. J..

[47] Duncan P.W., Weiner D.K., Chandler J., Studenski S. (1990). Functional reach: A new clinical measure of balance. J. Gerontol..

[48] Guralnik J.M., Simonsick E.M., Ferrucci L., Glynn R.J., Berkman L.F., Blazer D.G., Scherr P.A., Wallace R.B. (1994). A short physical performance battery assessing lower extremity function: Association with self-reported disability and prediction of mortality and nursing home admission. J. Gerontol..

[49] Álvarez-Gallardo I.C., Soriano-Maldonado A., Segura-Jiménez V., Carbonell-Baeza A., Estévez-López F., McVeigh J.G., Delgado-Fernández M., Ortega F.B. (2016). International FItness Scale (IFIS): Construct validity and reliability in women with fibromyalgia: The al-Ándalus Project. Arch. Phys. Med. Rehab..

[50] Fischer A.A. (1997). Algometry in the daily practice of pain management. J. Back Musculoskelet Rehabil..

[51] Boonstra A.M., Preuper H.R.S., Reneman M.F., Posthumus J.B., Stewart R.E. (2008). Reliability and validity of the visual analogue scale for disability in patients with chronic musculoskeletal pain. Int. J. Rehabil. Res..

[52] Kersten P., White P.J., Tennant A. (2014). Is the pain visual analogue scale linear and responsive to change? An exploration using Rasch analysis. PLoS ONE.

[53] Laas K., Roine R., Räsänen P., Sintonen H., Leirisalo-Repo M., Group H.Q.S. (2009). Health-related quality of life in patients with common rheumatic diseases referred to a university clinic. Rheumatol. Int..

[54] Sintonen H. (2001). The 15D instrument of health-related quality of life: Properties and applications. Ann. Med..

[55] Vilagut G., Valderas J., Ferrer M., Garin O., López-García E., Alonso J. (2008). Interpretation of SF-36 and SF-12 questionnaires in Spain: Physical and mental components. Med. Clin..

[56] Ruiz M., Rejas J., Soto J., Pardo A., Rebollo I. (2003). Adaptation and validation of the Health Utilities Index Mark 3 into Spanish and correction norms for Spanish population. Med. Clin..

[57] Snyder-Halpern R., Verran J.A. (1987). Instrumentation to describe subjective sleep characteristics in healthy subjects. Res. Nurs. Health.

[58] Lines C.R., Vandormael K., Malbecq W. (2001). A comparison of visual analog scale and categorical ratings of headache pain in a randomized controlled clinical trial with migraine patients. Pain.

[59] Esteve-Vives J., Redondo J.R., Salvat M.I.S., de Gracia Blanco M., de Miquele C.A. (2007). Proposal for a consensus version of the Fibromyalgia Impact Questionnaire (FIQ) for the Spanish population. Reumatol. Clin. (Engl. Ed.).

[60] Luciano J.V., Aguado J., Serrano-Blanco A., Calandre E.P., Rodriguez-Lopez C.M. (2013). Dimensionality, reliability, and validity of the revised fibromyalgia impact questionnaire in two Spanish samples. Arthritis Care Res..

[61] Salgueiro M., García-Leiva J.M., Ballesteros J., Hidalgo J., Molina R., Calandre E.P. (2013). Validation of a Spanish version of the revised fibromyalgia impact questionnaire (FIQR). Health Qual. Life Outcomes.

[62] Arnau R.C., Meagher M.W., Norris M.P., Bramson R. (2001). Psychometric evaluation of the Beck Depression Inventory-II with primary care medical patients. J. Health Psychol..

[63] Steer R.A., Clark D.A., Beck A.T., Ranieri W.F. (1999). Common and specific dimensions of self-reported anxiety and depression: The BDI-II versus the BDI-IA. Behav. Res. Ther..

[64] Lyubomirsky S., Lepper H.S. (1999). A measure of subjective happiness: Preliminary reliability and construct validation. Soc. Indic. Res..

[65] Diener E., Emmons R.A., Larsen R.J., Griffin S. (1985). The satisfaction with life scale. J. Pers..

[66] Munguía-Izquierdo D., Legaz-Arrese A., Mannerkorpi K. (2011). Transcultural adaptation and psychometric properties of a Spanish-language version of physical activity instruments for patients with fibromyalgia. Arch. Phys. Med. Rehab..

[67] Munguia-Izquierdo D., Segura-Jimenez V., Camiletti-Moiron D., Alvarez-Gallardo I., Estevez-Lopez F., Romero A., Delgado-Fernandez M. (2013). Spanish adaptation and psychometric properties of the Sedentary Behaviour Questionnaire for fibromyalgia patients: The al-Andalus study. Clin. Exp. Rheumatol..

[68] Rosenberg D.E., Norman G.J., Wagner N., Patrick K., Calfas K.J., Sallis J.F. (2010). Reliability and validity of the Sedentary Behavior Questionnaire (SBQ) for adults. J. Phys. Act. Health.

[69] Turk D.C., Dworkin R.H., Allen R.R., Bellamy N., Brandenburg N., Carr D.B., Cleeland C., Dionne R., Farrar J.T., Galer B.S. (2003). Core outcome domains for chronic pain clinical trials: IMMPACT recommendations. Pain.

[70] López Bastida J., Oliva J., Antoñanzas F., García-Altés A., Gisbert R., Mar J., Puig-Junoy J. (2010). Propuesta de guía para la evaluación económica aplicada a las tecnologías sanitarias. Gac. Sanit..

[71] Drummond M.F., Sculpher M.J., Claxton K., Stoddart G.L., Torrance G.W. (2015). Methods for the Economic Evaluation of Health Care Programmes.

[72] Flynn T.N., Peters T.J. (2005). Cluster randomized trials: Another problem for cost-effectiveness ratios. Int. J. Technol. Assess. Health Care.

[73] EUROSTAT (2015). People in the EU: Who Are We and How Do We Live?.

[74] Lutz W., Butz W.P., Samir K.E. (2014). World Population and Human Capital in the Twenty-First Century.

[75] Gusi N., Herrera E., Quesada F., Cebrian C., Juan C.C. (2008). Exercise looks after you: From research to practice in elderly. J. Aging Phys. Act..

